# The Optimal Strategy of Vitamin D for Sarcopenia: A Network Meta-Analysis of Randomized Controlled Trials

**DOI:** 10.3390/nu13103589

**Published:** 2021-10-14

**Authors:** Shih-Hao Cheng, Kee-Hsin Chen, Chiehfeng Chen, Woei-Chyn Chu, Yi-No Kang

**Affiliations:** 1 Department of Biomedical Engineering, National Yang Ming Chiao Tung University, Taipei 112, Taiwan; franchpaladin@gmail.com; 2 Department of Orthopedics, Cheng Hsin General Hospital, Taipei 112, Taiwan; 3 Department of Orthopedics, Wan Fang Hospital, Medical University Hospital, Taipei 116, Taiwan; 4 Post-Baccalaureate Program in Nursing, College of Nursing, Taipei Medical University, Taipei 110, Taiwan; keehsin@tmu.edu.tw; 5 Cochrane Taiwan, Taipei Medical University, Taipei 110, Taiwan; clifchen@tmu.edu.tw; 6 Center for Nursing and Healthcare Research in Clinical Practice Application, Wan Fang Hospital, Taipei Medical University, Taipei 116, Taiwan; 7 Evidence-Based Knowledge Translation Center, Wan Fang Hospital, Taipei Medical University, Taipei 116, Taiwan; 8 Evidence-Based Medicine Center, Wan Fang Hospital, Medical University Hospital, Taipei 116, Taiwan; 9 Division of Plastic Surgery, Department of Surgery, Wan Fang Hospital, Taipei Medical University, Taipei 116, Taiwan; 10 Department of Public Health, School of Medicine, College of Medicine, Taipei Medical University, Taipei 110, Taiwan; 11 Research Center of Big Data and Meta-Analysis, Wan Fang Hospital, Taipei Medical University, Taipei 116, Taiwan; 12 Institute of Health Policy & Management, College of Public Health, National Taiwan University, Taipei 100, Taiwan

**Keywords:** muscle mass, grip strength, gait speed, sarcopenia, vitamin D

## Abstract

Sarcopenia is a disease of gradual loss of muscle mass in elderly people, and the most common treatment options include nutritional supplementation and exercise. Vitamin D has potential beneficial effects for skeletal muscle tissue and has often been included in nutritional therapy formulations. However, the therapeutic effect of vitamin D for the treatment of sarcopenia has not yet been determine and there is a lack of high-quality supporting evidence. We searched three databases for randomized controlled trials (RCTs) on this topic. Changes in hand grip strength, gait speed, chair-stand test, fat mass, relative skeletal muscle index, and muscle mass were assessed for analysis. Network meta-analysis was further employed, based on the frequentist approach. Outcomes were reported as weighted mean differences (WMD) with 95% confidence intervals (CIs). A total of 9 RCTs (*n* = 1420) met our eligibility criteria, which treated patients with vitamin D (D), protein (P, *n* = 165), exercise (E, *n* = 124), iso-caloric product (I, *n* = 226), usual care without nutritional supplement (*n* = 65), P + D (*n* = 467), D + E (*n* = 72), P + E (*n* = 69), D + E + I (*n* = 73), and P + D + E (*n* = 159). The pooled estimate showed that the P + D + E intervention induced a greater improvement in hand grip strength than iso-caloric product intervention (WMD = 3.86; 95%CI, 0.52–7.21). Vitamin D intervention could lead to shorter chair-stand time (WMD = −1.32; 95%CI, −1.98 to −0.65), but no significant findings could be found for gait speed and muscle mass outcomes. Our synthesis found that combining vitamin D supplementation with protein supplementation and exercise can significantly increase grip strength and also showed a trend toward increasing muscle mass. This result implies that adding vitamin D to a standard treatment protocol for sarcopenia may be helpful for regaining function.

## 1. Introduction

Sarcopenia is an aging process involving loss of skeletal muscle mass in elderly people [1]. In 2010, the European Working Group of Sarcopenia in Older People (EWGSOP) published a definition and consensus [2]. This definition has since been used worldwide, and research into sarcopenia has increased. In consideration of the differences between Asian and Caucasian populations, the Asian Working Group for Sarcopenia published a diagnostic diagram based on Asian data [3]. In 2018, the EWGSOP updated their definition and modified their guidelines [4]. Sarcopenia is now generally accepted as a disease entity rather than a physiological change that occurs with aging, and it has a diagnostic code in the International Classification of Diseases, Tenth Revision, Clinical Modification (ICD-10-CM), requiring treatment.

Although many studies have discussed treatment of sarcopenia, there are no standard guidelines for management of the disease. Ongoing trials are attempting to develop therapies targeting myostatin and the activin receptor. Hormone therapies are also being researched. However, there is no US Food and Drug Administration-approved drug for the treatment of sarcopenia. Currently, the main evidence-based therapies are physical exercise programs and nutritional supplementation. However, there is no consensus on the duration, intensity, and protocol of exercise programs. The regimen and dosage for nutritional supplementation are also unclear. Most studies have included vitamin D in their treatment protocols, based on its hypothesized positive effects on muscle systems. Thus, the efficacy of vitamin D treatment deserves more discussion.

Understanding the role of vitamin D in the human body has evolved over recent decades. Its role in treating osteoporosis is well established, and regular supplementation is recommended in patients who receive osteoporotic treatment [5]. Vitamin D suppresses the expression of myostatin in muscle tissue, which is an inhibitor of muscle growth [6]. Theoretically, suppression of myostatin leads to muscle proliferation. Vitamin D also protects the skeletal muscle from acute damage [7]. Many cross-sectional studies have revealed a relationship between vitamin D deficiency and decreased physical function and reduced muscle mass and grip strength [8,9]. However, most of these studies indicated that their results may be confounded by underlying malnutrition. The therapeutic effect of vitamin D is still controversial. Some meta-analyses have shown that vitamin D may have positive effects on lower-limb muscle power [10], whereas others showed vitamin D to have only weak effect, or even no effect, on increasing muscle mass or strength [11]. It is noteworthy that these studies did not focus on populations with pre-existing sarcopenia. Currently, the involvement of vitamin D in nutritional therapy for sarcopenia is based on consensus, not evidence. In the present network meta-analysis, we analyzed randomized controlled trials (RCTs) that discuss the use of vitamin D in treating sarcopenia. To the best of our knowledge, this is the first comprehensive review to use network meta-analysis to investigate vitamin D supplementation for the treatment of sarcopenia.

## 2. Methods

The procedures and reporting of this work adhere to the Preferred Reporting Items for Systematic Reviews and Meta-Analyses (extension for network meta-analysis). The primary inclusion criteria for studies were as follows: (a) enrolled patients with sarcopenia, (b) randomly allocated patients in intervention groups, and (c) patients treated with vitamin D. The exclusion criteria were as follows: (a) recruited patients with pre-sarcopenia, (b) were not fully published (gray literature without details regarding methods and findings), and (c) focused on medication.

### 2.1. Data Sources and Evidence Selection

We searched Cochrane library and Cochrane CENTRAL, Embase, PubMed (with MEDLINE), and Web of Science to identify potential references using relevant keywords for sarcopenia and vitamin D. Boolean “OR” was used to increase sensitivity (e.g., “vitamin D” OR “vitamin D3” OR “vitamin D2” OR “ergocalciferol derivative” OR “dihydrotachysterol” OR “25 hydroxyvitamin D” OR “colecalciferol derivative”). Boolean “AND” was used to identify intersections of searches for sarcopenia and vitamin D. The search strategy did not use filters to restrict references to any specific publication year, patient age or sex, journal category, study design, or language. The search strategy was primarily built using PubMed; the other databases were also searched based on this strategy. The final search was performed for references before September 2021 (Supplementary Text S1).

Potential references were exported from the databases and imported into EndNote X9 software (Clarivate Analytics, Philadelphia, PA, USA) by two reviewers. Further screening was performed in two steps. In the first step, titles and abstracts were screened based on the eligibility criteria. Then, full texts were retrieved and reviewed independently by two reviewers. If full texts met any exclusion criteria, they were removed. If the two reviewers were not consistent in their selection, a final decision was made by the full review team through discussion and voting.

### 2.2. Data Extraction

While selecting studies, the two reviewers also extracted further data and performed double-checks. They used Excel software (Microsoft, Redmond, WA, USA) to record the surname of the first author, trial name, publication year, country, intervention of each group, age, sex, duration, and outcome data. Supplementations of protein and vitamin D in the present synthesis referred to extra dose of protein (overall protein ≥ 20 g or essential amino acid ≥ 3 g) and 800 IU vitamin D as a cut-off value [12]. Outcomes included both functional and body compositional data, including hand grip strength, gait speed, chair-stand test, body fat mass, relative skeletal muscle index (RSMI), as well as muscle mass of appendicular, lower extremity, and upper extremity. The functional outcomes were hand grip strength, gait speed, and chair-stand test. The body compositional outcomes consisted of muscle mass, RSMI, and body fat mass. Since these outcomes were continuous variables, the reviewers extracted means, measures of dispersion, and sample sizes of each group. Measures of dispersion included standard deviation, standard error (SE), interquartile range, and 95% confidence interval (CI).

### 2.3. Quality Evaluation

Quality evaluation was performed according to the main concepts of risk of bias 2 (RoB 2), considering biases due to the randomization process, intended interventions, missing outcome data, measurement of outcomes, and selection of the reported result [13]. In accordance with RoB 2, the reviewers performed quality evaluation for each outcome and reported the overall risk of bias based on the worst-case scenario. Thus, an overall judgment of “high risk of bias” was made if any bias was deemed high risk, and “some concerns of risk of bias judgment” if any bias raised some concerns without being high risk. If the two reviewers were inconsistent in their RoB 2 evaluation, the final decision was made by the full review team through discussion and voting.

### 2.4. Data Synthesis and Analysis

We tabulated qualitative information to obtain an overview of the characteristics of the trials. To test the effectiveness of vitamin D for sarcopenia, we performed further network meta-analysis using the frequentist approach. We based the planned meta-analysis on data of difference in differences. However, most trials did not report score changes for muscle mass. Therefore, we used data of changes for hand grip, gait speed, RSMI, and body fat mass; we use only data from final observations of appendicular, lower extremity, and upper extremity muscle mass. The pooled effects of vitamin D for all outcomes were presented as weighted mean differences (WMD) with corresponding 95% CI, because of the similarity of outcome measurements across the included trials. Moreover, we further calculated *P*-scores to clarify the optimal strategy for vitamin D intervention in sarcopenia. The *P*-score is a statistical technique to show the means of one-sided *p*-values, using point estimates and SEs, in a network meta-analysis using the frequentist approach. When the *P*-score of an intervention strategy in a specific outcome is close to 1, that strategy is indicated as a better intervention for the outcome, among all the intervention strategies.

The appropriateness of pooled estimates can be evaluated using tests of inconsistency and publication bias. Our inconsistency test was based on the design-by-treatment interaction model, since two-arm and four-arm RCTs contributed to the meta-analysis. Publication bias was explored using adjusted funnel plots. If data were sufficient, an Egger’s test of the intercept was further performed, using centralized effect size with SE.

Since data for the chair-stand test were only available from two trials (comparing the combination of vitamin D and protein to usual care), a consistency model was unnecessary. Data on the chair-stand test were pooled using head-to-head meta-analysis in a random-effects model; heterogeneity across RCTs was presented as an I-square index. According to the common threshold for determination of heterogeneity, the pooled estimate of the chair-stand test would be heterogeneous if I-square is greater than 50%. All of the abovementioned analyses were carried out using R software version 4.0.3 (www.r-project.org) using the netmeta, netrank, funnel.netmeta, and metacont functions. If a consistency model was a complete network with significant findings, further confidence rating would be evaluated [14].

## 3. Results

**Figure 1:**
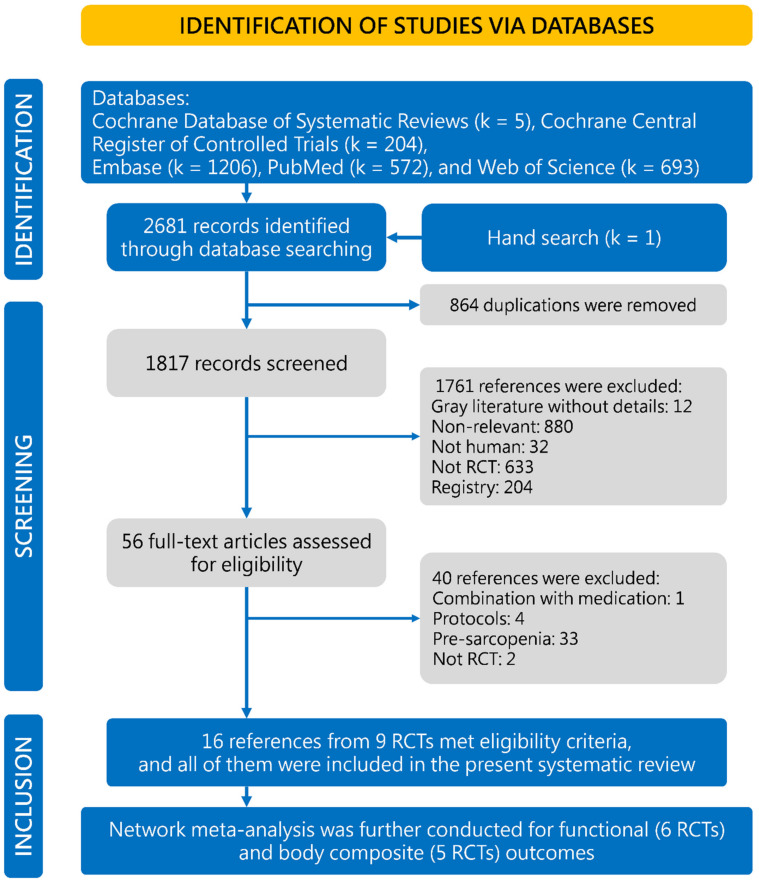
Flowchart of this synthesis. RCT, randomized clinical trial.

We identified 2681 references, comprising 2681 from Cocrhane database (k = 5), Cochrane CENTRAL Register of Controlled Trials (k = 204), Embase (k = 1206), PubMed (k = 572), and Web of Science (k = 693), plus 1 reference identified in the reference lists of relevant RCTs. We used Endnote functions to remove duplicates (k = 786), and further manually removed duplicates when Endnote did not detect them (k = 76). Then, 1763 records were routed out due to irrelevant (k = 880), not human (k = 32), not RCT (k = 634), and gray literature without details (k = 12). Full texts of the 56 remaining references were retrieved for eligibility review. Finally, 16 references published for 9 RCTs met our eligibility criteria, after 40 references were excluded due to their protocols (k = 4), not RCT (k = 2), combination of medication (k = 1), and pre-sarcopenia (k = 33). All references of the nine RCTs were included (Figure 1) [15,16,17,18,19,20,21,22,23,24,25,26,27,28,29,30].

### 3.1. Characteristics and Quality of Included Studies

We included 9 RCTs with 1420 patients with sarcopenia from 2015 and 2020. Although many trials had wished to investigate the effects of vitamin D, no trial provided patients with only vitamin D at a therapeutic dose. In the included trials, vitamin D was commonly combined with protein or exercise. A total of 9 categories of intervention could be found in the included RCTs, including protein only (P, *n* = 165), exercise only (E, *n* = 124), iso-caloric product only (I, *n* = 226), usual care without nutritional supplement (*n* = 65), P + D (*n* = 467), D + E (*n* = 72), P + E (*n* = 69), D + E + I (*n* = 73), and P + D + E (*n* = 159). The minimum age in each trial ranged from 60 to 74 years, according to the available information. Female rate in most trials were higher than 50%, except for the FrOST trial. Most of the included trials recruited patients with non-deficient of vitamin D at baseline. Further information is presented in Table 1. Risk of bias is shown in Supplementary Table S1.

### 3.2. Functional Outcomes

**Figure 2:**
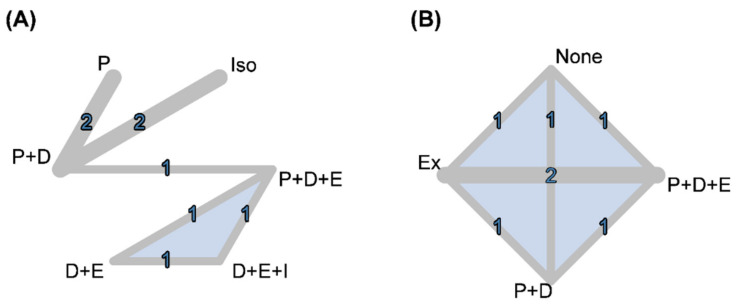
Network graphs of (**A**) main functional outcome (changes in hand grip) and (**B**) main body compositional outcome (muscle mass). D, vitamin D; E/Ex, exercise; Iso, iso-caloric product; P, protein.

**Figure 3:**
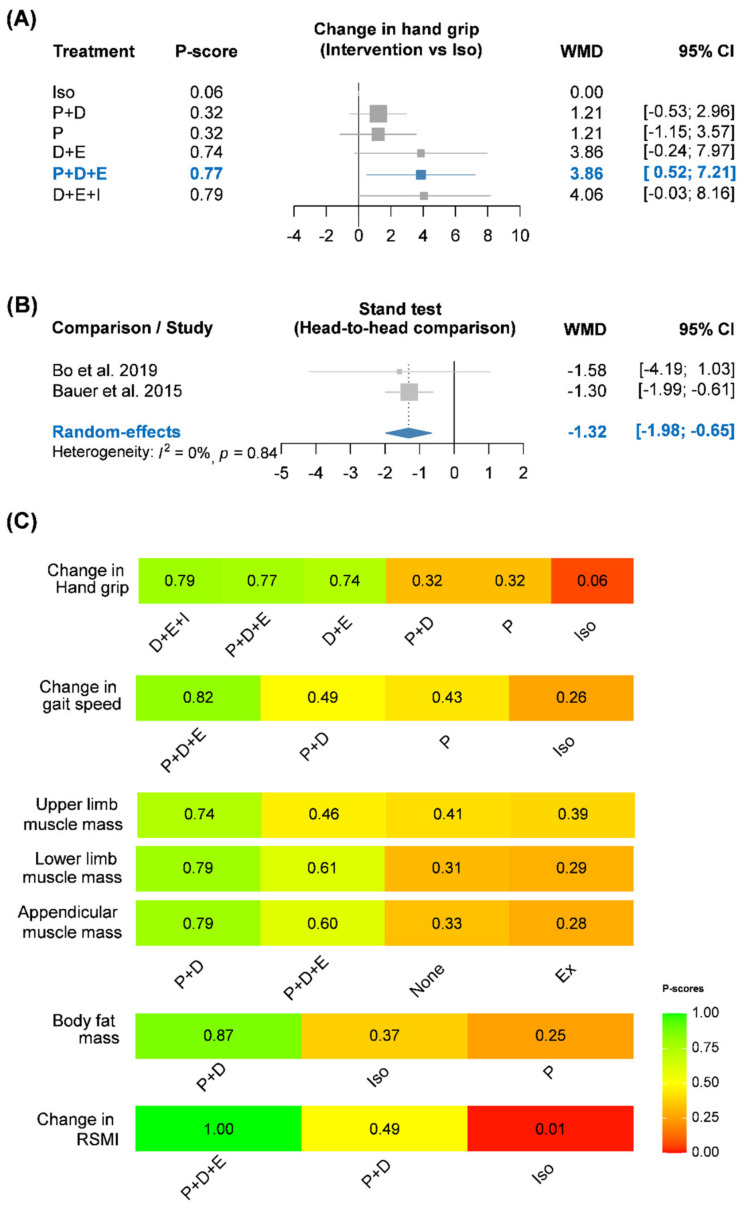
Forest plots of (**A**) change in hand grip and (**B**) time to chair-stand test, and (**C**) rainbow plot of P-scores. CI, confidence interval; D, vitamin D; E/Ex, exercise; Iso, iso-caloric product; P, protein; RSMI, relative skeletal muscle index; WMD, weighted mean difference.

The functional outcomes were changes in hand grip strength, gait speed, and time to chair-stand. Six RCTs presented data on changes in hand grip strength. Only five contributed to a six-node network meta-analysis of changes in hand grip strength (Figure 2) [16,17,18,20,22,23]; the other trial was disconnected from the network [28]. The network involved 1029 sarcopenia cases treated by P only (*n* = 165), I only (*n* = 226), vitamin D (D) + P (*n* = 399), D + E (*n* = 72), D + E + I (*n* = 73), and P + D + E (*n* = 94). Pooled results (with usual care as the reference) showed that P + D + E (WMD, 3.86; 95% CI, 0.52–7.21) had significantly greater hand grip strength. Although P, P + D, D + E, and D + E + I did not have statistical significance, the effect sizes of D + E + I (WMD, 4.06; 95% CI, −0.03–8.16) and D + E (WMD, 3.86; 95% CI, −0.24–7.97) may reach clinical significance. No significant difference was found among all of the protein treatments (P + D, D + E, D + E + I, and P + D + E). The *P*-score results indicated that interventions involving vitamin D and exercise were better among the five active treatments (Figure 3). Using design-by-treatment interaction model, inconsistency was not detected in the network meta-analysis of hand grip strength (Supplementary Text S2). No serious asymmetry appeared in a comparison-adjusted funnel plot.

The second functional outcome was change in gait speed. A total of 3 RCTs formed a 4-node network meta-analysis (Supplementary Figure S1) [16,20,22,23] involving 751 sarcopenia cases treated with I (*n* = 196), P (*n* = 165), P + D (*n* = 369), and P + D + E (*n* = 21). Although no significant difference existed among the four groups (Table 2), the pooled estimates provided meaningful trends regarding their combination effect. For instance, protein alone had the least effect size, and P + D + E had the greatest effect size. *P*-scores were calculated to analyze changes in gait speed among the four treatment strategies (Figure 3). P + D + E obtained the highest *P*-score for improving gait speed (*P*-score = 0.82), followed by P + D (*P*-score = 0.49) and protein (*P*-score = 0.43). Since there was no loop in this network, an inconsistency test could not be performed. No serious asymmetry appeared in a comparison-adjusted funnel plot of changes in gait speed.

The final functional outcome was time to chair-stand. Relevant data were available only for comparison of usual care with the combination of vitamin D and protein in two RCTs (*n* = 324) [16,18]. The pooled result showed that patients after combination treatment had greater reduction in time to chair-stand test than those with usual care (WMD, −1.32; 95% CI, −1.98 to −0.65). Heterogeneity in the pooled estimate of time to chair-stand was very low across RCTs (I-square = 0%; *p* > 0.10; Figure 3).

### 3.3. Body Compositional Outcomes

**Figure 4:**
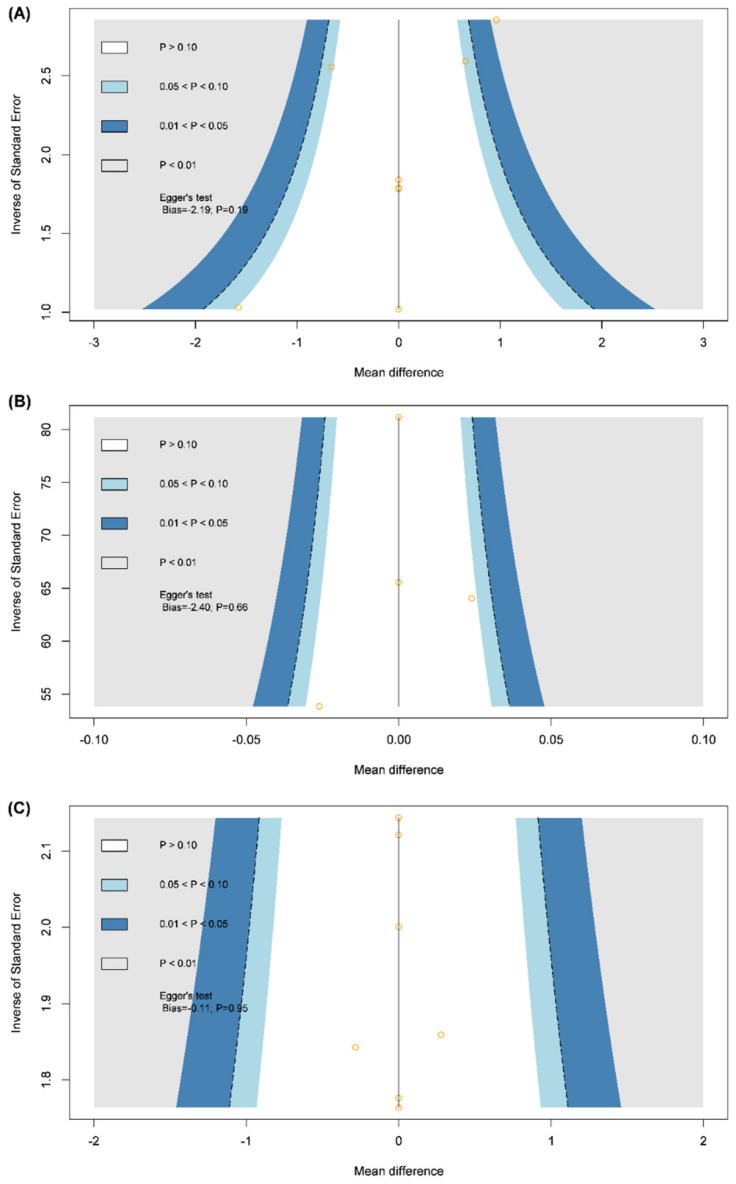
Funnel plots of (**A**) change in hand grip, (**B**) change in gait speed, and (**C**) appendicular muscle mass.

The structural outcomes included: muscle mass, change in RSMI, and change in body fat mass. Relevant data on muscle mass were available for a four-node network meta-analysis, including E, P + D, P + D + E, and usual care [19,25]. Pooled estimates did not show any statistically significant difference in all analyses of muscle mass among the four groups (Supplementary Figure S2), but groups with vitamin D intervention had higher *P*-scores: P + D and P + D + E were ranked highest for appendicular muscle mass, lower-limb muscle mass, and upper-limb muscle mass (Figure 3). The design-by-treatment model did not detect inconsistency, and a comparison-adjusted funnel plot appeared to be symmetric (Figure 4). With regard to change in RSMI, only two RCTs had available data to form a three-node network model, including iso-caloric product, P + D, and P + D + E [18,22,23]. Pooled estimates showed that P + D + E (WMD, 0.52; 95% CI, 0.33–0.71) and P + D (WMD, 0.17; 95% CI, 0.01–0.33) significantly improved in RSMI compared to the iso-caloric product (Table 2). Three RCTs reported data on change in body fat mass appropriately [18,20], and the data could form a three-node consistency model without a loop. The network consisted of iso-caloric product, protein, and P + D. Pooled estimates showed that no significant difference among the tree intervention groups, while P + D also had the highest *P*-score (0.87). Since there was no loop in the network models of changes in RSMI and body fat mass, no inconsistency tests could be performed. Funnel plots of the two outcomes are shown in Supplementary Figures S3 and S4.

## 4. Discussion

### 4.1. Key Findings

Our review synthesized a total of 9 RCTs and 1420 patients. Network meta-analysis revealed that combining vitamin D supplementation with exercise and protein supplementation can increase grip strength. Combination of vitamin D and protein could shorten time to chair-stand. For gait speed and lower-limb mass, vitamin D supplementation, either alone or combined with other treatments, showed a trend of beneficial effect, yet it did not reach statistical significance.

Vitamin D plays an important role in maintaining the physiological function of skeletal muscle. Although the specific mechanism for this is still unclear, several possible hypotheses have been proposed. Studies have demonstrated that vitamin D receptor (VDR) is found in skeletal muscle cells, and that vitamin D has an extensive effect on muscle tissue [31]. By binding with VDR in the nucleus, vitamin D may inhibit the expression of myostatin, which is an inhibitor of muscle growth [32]. Thus, suppression of myostatin increases muscle cell proliferation [33]. Vitamin D also plays an important role in calcium and inorganic phosphate metabolism, both of which are important for muscle contractility [32]. The positive effects of vitamin D on the muscle have been demonstrated in previous trials. Several RCTs have reported that vitamin D (alone or in combination therapy) can effectively elevate muscle power and function in elderly people [12,30], whereas other studies have shown that vitamin D supplementation cannot elevate muscle mass or increase physical function [34]. Our synthesis echoed the positive effects of vitamin D on skeletal muscle. In combination with exercise and protein supplementation, it can significantly improve grip strength. For gait speed and muscle mass, our results showed a trend toward better treatment effect but did not meet statistical significance. There are several possible explanations for our results. Firstly, the effect size of vitamin D supplementation may be small, and our case numbers may not be sufficient to reveal any influence. Secondly, there is currently no standard therapeutic dose of vitamin D for the treatment of sarcopenia; we used a common dose of 800 IU as a cut-off value [12]. However, some enrolled studies did not measure baseline vitamin D levels before intervention. For patients with normal vitamin D levels, supplementation of vitamin D may have no effect. However, for patients with severe underlying vitamin D deficiency, a dosage of 800 IU daily may not be sufficient [35]. According to existing data, a majority of the population has vitamin D deficiency, especially women and elderly people [36]. Elderly people with vitamin D deficiency may have concomitant malnutrition [37]; simple vitamin D supplementation may not obtain satisfactory results.

When vitamin D was added in treatment, we observed a trend of increasing muscle mass (whole body and lower limbs). Exercise appeared not to have significant effects for increasing muscle mass, which runs counter to common knowledge. One reason for this may be that exercise is not easily quantified; there exist many heterogeneities in exercise treatment, such as duration, intensity, trained or untrained, and training protocol. Furthermore, elderly people may have poor compliance to exercise treatment. Patients with sarcopenia may not cooperate with an intense training program, while easier exercise may not reach the intensity necessary for increasing muscle mass [38]. Our finding is similar to the results of previous reviews, which have shown no obvious effect of exercise therapy for increasing muscle mass [39].

### 4.2. Limitations

Our review has several limitations. Firstly, the number of studies and cases is limited; more high-quality RCTs are needed to confirm the efficacy of vitamin D supplementation. Furthermore, the diagnostic criteria for sarcopenia are still changing, and they are different between races. The studies we analyzed were conducted in different regions, and patients were diagnosed using different criteria and tools. Since a diagnosis of “pre-sarcopenia” is not included in the existing guidelines, we excluded all patient groups with this diagnosis. Therefore, our analysis can explain only the use of vitamin D for treating patients with sarcopenia, not for preventing sarcopenia. The included trials recruited patients using various criteria for defining sarcopenia, and the inconsistency across the RCTs might threat the quality of the current synthesis. All of the articles we included reported results without differentiating sex. However, baseline and diagnostic cut-off values are different between male and female. The same effect size represents a different ratio of change in different sexes and deserves to be discussed separately. Another concern is the form of vitamin D supplementation. The absorption ratio of vitamin D differs between D2 and D3 and may also be affected by different dosage forms. Many of the trials in this review did not precisely indicate the form of vitamin D used in treatment. In other words, the insufficient information on the dosage of vitamin D and the duration of treatment limit the understanding of the use of vitamin D for sarcopenia since no further analysis could be carried out due to the incomplete data in the included RCTs. The final issue must be addressed before application of vitamin D in sarcopenia management is that effects of vitamin D may relate to sun exposure, but this factor might be not controlled well in the included studies. Appropriate patient education regarding vitamin D supplementation and sun exposure ought to be taken into consideration in clinical practice and further studies in the future.

## 5. Conclusions

Vitamin D supplementation for patients with sarcopenia may significantly improve grip strength, when combined with exercise and protein supplementation. In addition, vitamin D supplementation showed a trend toward increasing muscle mass and function, yet this did not reach statistical significance. Current evidence for supplementation with vitamin D alone is not strong, while vitamin D in patients with sarcopenia could be a routine supplementation due to safety and not high cost. This evidence could be applied to those with mild deficiency and non-deficiency of vitamin D at baseline. Although there is a great deal of research ongoing regarding pharmacologic treatments for sarcopenia, there are still no FDA-approved medicines to treat the disease; yet, vitamin D supplementation, exercise programs, and nutritional treatment show promising results, the effect sizes are not satisfactory. For greater therapeutic effects, we must give more emphasis to the development of medications, to be used in conjunction with the aforementioned treatments. 

## Figures and Tables

**Table 1:** Characteristics of the included randomized controlled trials.

				**Criteria for** **Sarcopenia**	**Type of** **Sarcopenia**	**Intervention** **Duration**
**Author**	**Year**	**Area**	**Age**			
PROVIDE trial	2015–2020	Europe	≥65	SBBP 4~9 SMI < 37%/28%	Primary	13 weeks
Cramer	2016	Europe + America	≥65	EWGSOP	Primary	24 weeks
Kim	2016	Asia (Japan)	≥70	SMI < 5.67 kg/m^2^	Primary	3 months
				Grip strength < 17 kg		
				Walk speed < 1 m/s		
Rondanelli	2016	Europe (Italy)	≥65	Relative muscle mass	Primary	12 weeks
				<7.26/5.5 kg/m^2^ (M/F)		
Bo	2017	Asia (China)	60–85	AWGS	Primary	6 months
Takeuchi	2018	Asia (Japan)	≥65	AWGS	Primary	8 weeks
Björkman	2019	Europe (Finland)	≥74	Complicated	Primary	12 months
Chang	2020	Asia (Taiwan)	≥65	EWGSOP	Primary	12 weeks
FrOST trial (Kemmler)	2018–2020	Europe (Germany)	≥72	EWGSOP	Primary	18 months
					**Baseline Vitamin D**	
**Author**	**Intervention**			**Sex (M/F)**	**Serum Vitamin D**	**Deficiency**
PROVIDE trial	P + D (vitamin D 1600 IU/day, whey protein 40 g)			64/120	25(OH)D 48 (nmol/L)	Deficient
	Iso-caloric product			67/129	25(OH)D 49 (nmol/L)	Deficient
Cramer	P + D (vitamin D3 998 IU/day, protein 40 g)			63/102	Vitamin D 65 (nmol/L)	Non-deficient
	Protein (protein 28 g with non-therapeutic dose vitamin D3)			63/102	Vitamin D 60 (nmol/L)	Non-deficient
Kim	P + D + E (vitamin D 800 IU/day, leucine-enriched amino acid 3 g)			0/36	Vitamin D 23.2 (ng/mL)	Non-deficient
	P + D (vitamin D 800 IU/day, leucine-enriched amino acid 3 g)			0/34	Vitamin D 22.5 (ng/mL)	Non-deficient
	Exercise			0/35	Vitamin D 24.2 (ng/mL)	Non-deficient
	No nutritional supplement (with health education only)			0/34	Vitamin D 27.0 (ng/mL)	Non-deficient
Rondanelli	P + E (with non-therapeutic dose vitamin D3, essential amino acids 32 g)			29/40	Not reported	Not reported
	Exercise (with non-therapeutic dose vitamin D3)			24/37	Not reported	Not reported
Bo	P + D (vitamin D 1404 IU/day, protein 44 g)			13/17	Vitamin D3 21.29 (ng/mL)	Non-deficient
	Iso-caloric product			14/16	Vitamin D3 20.85 (ng/mL)	Non-deficient
Takeuchi	P + D (vitamin D 12.5 μg/day, BCAA 10 g)			12/20	Not reported	Not reported
	No nutritional supplement			13/18	Not reported	Not reported
Björkman	D + E (vitamin D 800 IU/day)			16/56	Not reported	Not reported
	D + E + I (vitamin D 800 IU/day, iso-caloric product)			27/46	Not reported	Not reported
	P + D + E (vitamin D 800 IU/day, whey protein 20 g)			22/51	Not reported	Not reported
Chang	Exercise			6/22	Not reported	Not reported
	P + D + E (vitamin D3 1600 IU/day, BCAA 6 g)			7/22	Not reported	Not reported
FrOST trial	P + D + E (vitamin D 2500–5000 IU/week, whey protein 80 g)			21/0	25(OH)D 21.6 (ng/mL)	Non-deficient
	P + D (vitamin D 2500–5000 IU/week, whey protein 80 g)			22/0	25(OH)D 17.5 (ng/mL)	Deficient

AWGS, Asian Working Group for Sarcopenia; BCAA, Branched-chain amino acids; D, vitamin D; E, exercise; EWGSOP, European Working Group on Sar-copenia in Older People; F, female; I, iso-caloric product; M, male; P, protein; S, selective androgen receptor modulator; SBBP, short physical performance battery; SMI, skeletal muscle index.

**Table 2:** League table of network meta-analysis findings. Mean difference (95% confidence interval).

**Changes in** **gait speed**	Iso.				Nil
	0.02 (−0.07,0.10)	P			
	0.02 (−0.05,0.09)	0 (−0.04,0.05)	P + D		
	0.05 (−0.04,0.14)	0.03 (−0.05,0.12)	0.03 (−0.04,0.10)	P + D + E	
**Appendicular** **muscle mass**	NNS				Nil
	0.04 (−0.8,0.88)	Ex			
	−0.5 (−1.48,0.48)	0.54 (−0.5,1.58)	P + D		
	−0.24 (−1.08,0.6)	0.28 (−0.47,1.03)	−0.26 (−1.3,0.79)	P + D + E	
**Lower limbs** **muscle mass**	NNS	0.01 (−0.23,0.24)	−0.1 (−0.39,0.19)	−0.01 (−0.28,0.26)	Upper limbs muscle mass
	0.02 (−0.65,0.68)	Ex	−0.11 (−0.39,0.18)	−0.02 (−0.27,0.24)	
	−0.4 (−1.13,0.33)	0.42 (−0.39,1.23)	P + D	0.09 (−0.23,0.41)	
	−0.21 (−0.87,0.45)	0.23 (−0.39,0.84)	−0.19 (−0.99,0.61)	P + D + E	
**Changes in** **body fat mass**	Iso.	−0.17 (−0.33,−0.01)	–	−0.52 (−0.71,−0.33)	Changes in RSMI
	0.03 (−1.1,1.15)	P	–	−0.35 (−0.45,−0.25)	
	−0.4 (−1.44,0.64)	−0.43 (−0.86,0.00)	P + D	–	
	–	–	–	P + D + E	

D, vitamin D; Ex, exercise; Iso, iso-caloric product; NA, not applicable; NNS, no nutritional supplement; P, protein; RSMI, relative skeletal muscle index.

## Data Availability

All data generated or analysed during this study are included in this published article.

## Supplementary Materials

The following are available online at https://www.mdpi.com/article/10.3390/nu13103589/s1, Supplementary Text S1: Search strategy (Primary search strategy), Supplementary Text S2: Inconsistency test of changes in hand grip, Table S1: Risk of bias, Figure S1: Network plot of gait speed, Figure S2: Network forest plot of muscle mass, Figure S3: Funnel plot of relative skeletal muscle index, Figure S4: Funnel plot of body fat mass.

## Abbreviations

CI	confidence interval
EWGSOP	European Working Group of Sarcopenia in Older People
RCT	randomized controlled trial
RoB	risk of bias
RSMI	relative skeletal muscle index
SE	standard error
WMD	weighted mean differences
