# Supplementary material for: The Optimal Strategy of Vitamin D for Sarcopenia: A Network Meta-Analysis of Randomized Controlled Trials
Source: Nutrients. 2021 Oct 14;13(10):3589. doi: 10.3390/nu13103589 (PMC8541573; doi:10.3390/nu13103589)

Nutrients

The optimal strategy of vitamin D for sarcopenia: A network meta-analysis of randomized controlled trials

(Supplementary File)

Authors:

Shih-Hao Cheng, M.D.[1,2,4], Kee-Hsin Chen, R.N., Ph.D. [4,5,6,7], Chiehfeng Chen, Prof., M.D., Ph.D. [5,8,9,10], Woei-Chyn Chu, Prof., Ph.D. [1], Yi-No Kang, consultant, M.A.[5,8,11,12]

Affiliations:

1. Department of Biomedical Engineering, National Yang Ming Chiao Tung University, Taipei, Taiwan
2. Department of Orthopedics, Cheng Hsin General hospital, Taipei, Taiwan
3. Department of Orthopedics, Wan Fang Hospital, Medical University Hospital, Taipei, Taiwan
4. Post-Baccalaureate Program in Nursing, College of Nursing, Taipei Medical University, Taipei, Taiwan
5. Cochrane Taiwan, Taipei Medical University, Taipei, Taiwan
6. Center for Nursing and Healthcare Research in Clinical Practice Application, Wan Fang Hospital, Taipei Medical University, Taipei, Taiwan.
7. Evidence-based Knowledge Translation Center, Wan Fang Hospital, Taipei Medical University, Taipei, Taiwan.
8. Evidence-Based Medicine Center, Wan Fang Hospital, Medical University Hospital, Taipei, Taiwan
9. Division of Plastic Surgery, Department of Surgery, Wan Fang Hospital, Taipei Medical University, Taipei, Taiwan
10. Department of Public Health, School of Medicine, College of Medicine, Taipei Medical University, Taipei, Taiwan
11. Research Center of Big Data and Meta-analysis, Wan Fang Hospital, Taipei Medical University, Taipei, Taiwan
12. Institute of Health Policy & Management, College of Public Health, National Taiwan University, Taiwan

Supplementary

Supplementary Text 1. Search strategy

Supplementary Table S1. Risk of bias

Supplementary Text 2. Inconsistency test of changes in hand grip

Supplementary Figure S1. Network plot of gait speed

Supplementary Figure S2. Network forest plot of muscle mass

Supplementary Figure S3. Funnel plot of relative skeletal muscle index

Supplementary Figure S4. Funnel plot of body fat mass

Primary search strategy

#1. sarcopenia
#2. sarcopenic
#3. #1 OR #2
#4. vitamin D
#5. vitamin D3
#6. ergocalciferol derivative
#7. dihydrotachysterol
#8. 25 hydroxyvitamin D
#9. colecalciferol derivative
#10. #4 OR #5 OR #6 OR #7 OR #8 OR #9
#11. #3 AND #10

Search details in Cochrane Database and Cochrane CENTRAL

(sarcopenia OR sarcopenic) in All Text AND (vitamin D OR vitamin D3 OR ergocalciferol derivative OR dihydrotachysterol OR 25 hydroxyvitamin D OR colecalciferol derivative) in All Text - (Word variations have been searched)

Search details in Embase

('sarcopenia'/exp OR sarcopenia OR 'sarcopenia' OR sarcopenic:ti,ab) AND ('vitamin d'/exp OR 'vitamin d' OR '24,25 dihydroxyvitamin d'/exp OR '24,25 dihydroxyvitamin d' OR '25 hydroxyvitamin d'/exp OR '25 hydroxyvitamin d' OR 'vitamin d, 25 hydroxy' OR 'colecalciferol derivative'/exp OR 'cholecalciferol derivative' OR 'colecalciferol derivative' OR 'vitamin d3 derivative' OR 'dihydrotachysterol'/exp OR '10, 19 dihydroercalciol' OR '9, 10 seco 5, 7, 22 ergostatrien 3beta ol' OR '9, 10 secoergosta 5, 7, 22 trien 3beta ol' OR 'a.t.10' OR 'antitanil' OR 'antitetanin' OR 'antitetanine' OR 'at 10' OR 'at-10' OR 'at10' OR 'atecen' OR 'calcamin' OR 'calcamine' OR 'calcinosefaktor' OR 'dht intensol' OR 'dichistrolum' OR 'dichysterol' OR 'dichystrol' OR 'dihydral' OR 'dihydrotachysterin' OR 'dihydrotachysterine' OR 'dihydrotachysterol' OR 'dihydrotachysterol 2' OR 'dihydrotachysterol 3' OR 'dikystrol' OR 'dygratyl' OR 'hytakerol' OR 'manipal' OR 'parterol' OR 'tachidon' OR 'tachysterol, dihydro' OR 'tachystin' OR 'tachystine' OR 'tachystol' OR 'tetilan' OR 'ergocalciferol derivative'/exp OR 'calciferol derivative' OR 'ergocalciferol derivative' OR 'vitamin d2 derivative' OR 'vitamin d3')

Supplementary Text 1

Search strategy (Primary search strategy)

Search details in PubMed

("sarcopenia"[MeSH Terms] OR "sarcopenia"[All Fields] OR "sarcopenic"[All Fields]) AND ("vitamin d"[MeSH Terms] OR "vitamin d"[All Fields] OR "ergocalciferols"[MeSH Terms] OR "ergocalciferols"[All Fields] OR ("cholecalciferol"[MeSH Terms] OR "cholecalciferol"[All Fields] OR ("vitamin"[All Fields] AND "d3"[All Fields]) OR "vitamin d3"[All Fields]) OR (("ergocalciferols"[MeSH Terms] OR "ergocalciferols"[All Fields] OR "ergocalciferol"[All Fields]) AND ("analogs and derivatives"[MeSH Subheading] OR ("analogs"[All Fields] AND "derivatives"[All Fields]) OR "analogs and derivatives"[All Fields] OR "derivatives"[All Fields] OR "derivable"[All Fields] OR "derivant"[All Fields] OR "derivants"[All Fields] OR "derivate"[All Fields] OR "derivated"[All Fields] OR "derivates"[All Fields] OR "derivation"[All Fields] OR "derivations"[All Fields] OR "derivative"[All Fields] OR "derive"[All Fields] OR "derived"[All Fields] OR "derives"[All Fields] OR "deriving"[All Fields])) OR ("dihydrotachysterol"[MeSH Terms] OR "dihydrotachysterol"[All Fields]) OR ("25 hydroxyvitamin d"[Supplementary Concept] OR "25 hydroxyvitamin d"[All Fields] OR "25 hydroxyvitamin d"[All Fields] OR "calcifediol"[MeSH Terms] OR "calcifediol"[All Fields]) OR (("cholecalciferol"[MeSH Terms] OR "cholecalciferol"[All Fields] OR "cholecalciferols"[All Fields] OR "colecalciferol"[All Fields]) AND ("analogs and derivatives"[MeSH Subheading] OR ("analogs"[All Fields] AND "derivatives"[All Fields]) OR "analogs and derivatives"[All Fields] OR "derivatives"[All Fields] OR "derivable"[All Fields] OR "derivant"[All Fields] OR "derivants"[All Fields] OR "derivate"[All Fields] OR "derivated"[All Fields] OR "derivates"[All Fields] OR "derivation"[All Fields] OR "derivations"[All Fields] OR "derivative"[All Fields] OR "derive"[All Fields] OR "derived"[All Fields] OR "derives"[All Fields] OR "deriving"[All Fields])))

Search details in Web of Science

TOPIC: (sarcopenia OR sarcopenic) AND TOPIC: (vitamin D OR vitamin D3 OR ergocalciferol derivative OR dihydrotachysterol OR 25 hydroxyvitamin D OR colecalciferol derivative) Timespan: All years. Indexes: SCI-EXPANDED, SSCI, A&HCI, ESCI.

Supplementary Table S1

Risk of bias

Outcome / Trial	Bias 1	Bias 2	Bias 3	Bias 4	Bias 5	Overall
**Hand grip**						High
Björkman	Low	Low	High	Low	Low	High
Bo	Low	Low	Low	Low	Low	Low
Cramer	Low	Low	Some concerns	Low	Low	Some concerns
FrOST	Some concerns	Some concerns	Low	Low	Low	Some concerns
PROVIDE	Low	Low	Some concerns	Low	Low	Some concerns
**Gait speed**						Some concerns
Cramer	Low	Low	Some concerns	Low	Low	Some concerns
FrOST	Some concerns	Some concerns	Low	Low	Low	Some concerns
PROVIDE	Low	Low	Some concerns	Low	Low	Some concerns
**Chair-stand**						Some concerns
Bo	Low	Low	Low	Low	Low	Low
PROVIDE	Low	Low	Some concerns	Low	Low	Some concerns
**Relative skeletal muscle index**						Some concerns
Bo	Low	Low	Low	Low	Low	Low
FrOST	Some concerns	Some concerns	Low	Low	Low	Some concerns

Bias 1, randomization process; bias 2, intended interventions; bias 3, missing outcome data; bias 4, measurement of outcomes; bias 5, selection of the reported result

Supplementary Text 2

Inconsistency test of changes in hand grip

Q statistic to assess consistency under the assumption of a full design-by-treatment interaction random effects model

	Q	df	p-value	tau.within	tau2.within
Between designs	2.14	1	0.1439	1.0755	1.1567

Supplementary Figure S1

Network plot of gait speed

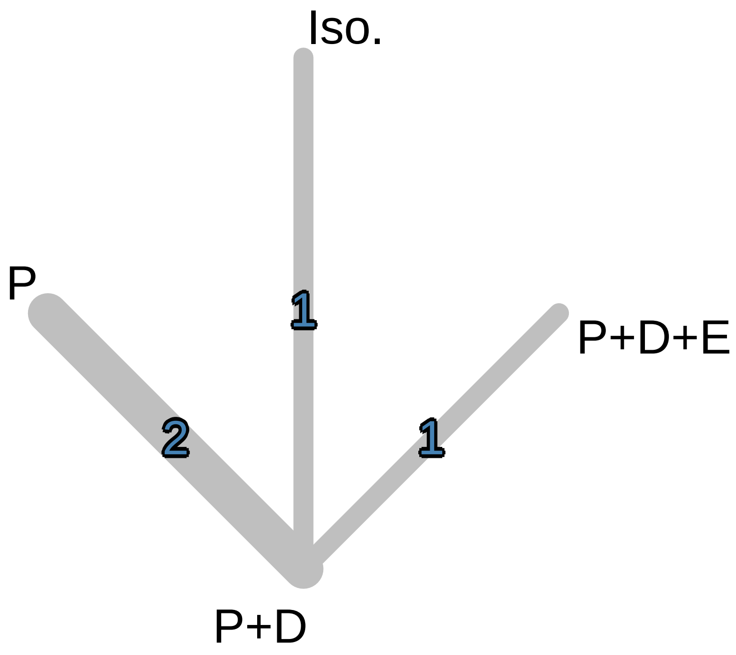

Supplementary Figure S2

Network forest plot of muscle mass

Appendicular muscle mass (Intervention vs None)

Treatment	P-score	WMD	95% CI
Ex	0.28	-0.04	[-0.88; 0.80]
None	0.33	0.00	
PDE	0.60	0.24	[-0.60; 1.08]
PD	0.79	0.50	[-0.48; 1.48]

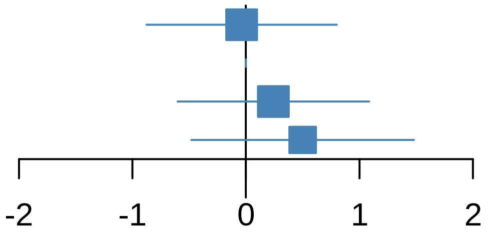

Upper limb muscle mass (Intervention vs None)

Treatment	P-score	WMD	95% CI
Ex	0.39	-0.01	[-0.24; 0.23]
None	0.41	0.00	
PDE	0.46	0.01	[-0.26; 0.28]
PD	0.74	0.10	[-0.19; 0.39]

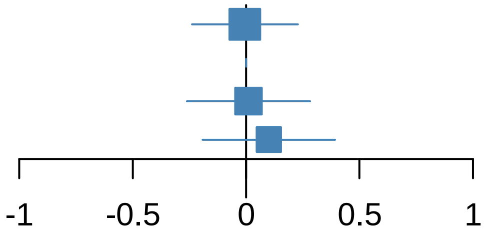

Lower limb muscle mass (Intervention vs None)

Treatment	P-score	WMD	95% CI
Ex	0.29	-0.02	[-0.68; 0.65]
None	0.31	0.00	
PDE	0.61	0.21	[-0.45; 0.87]
PD	0.79	0.40	[-0.33; 1.13]

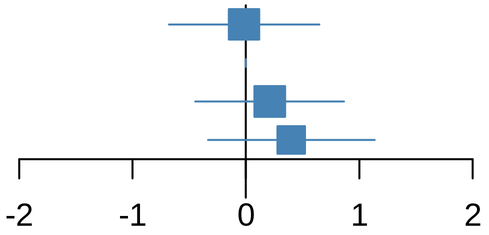

Supplementary Figure S3

Funnel plot of relative skeletal muscle index

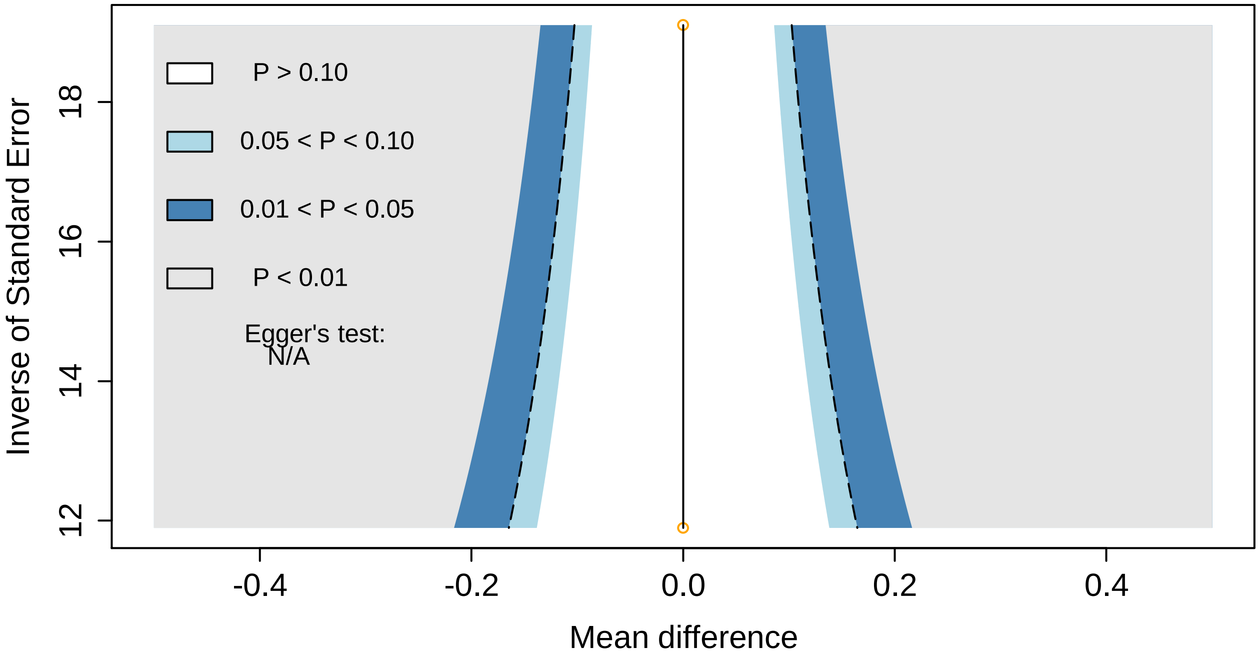

Supplementary Figure S4

Funnel plot of body fat mass

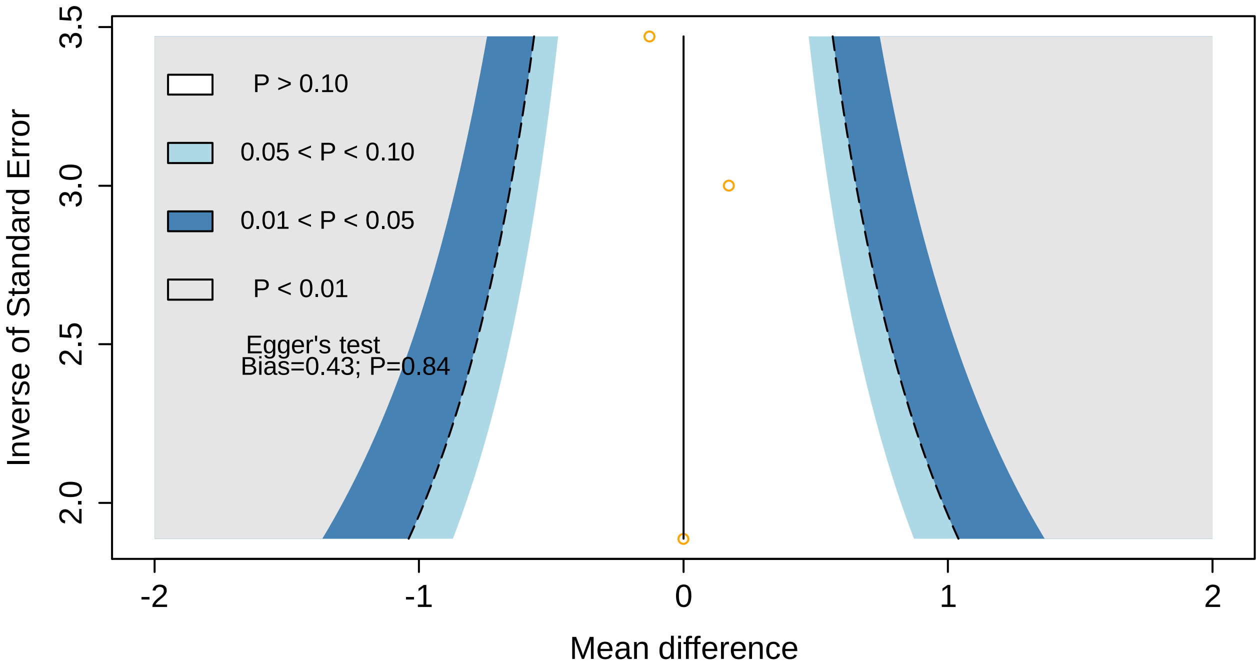

Supplement: Supplementary file 1 [file nutrients-13-03589-s001.zip › nutrients-1388560-supplementary.pdf]
